# The stress hyperglycemia ratio is associated with the development of cerebral edema and poor functional outcome in patients with acute cerebral infarction

**DOI:** 10.3389/fnagi.2022.936862

**Published:** 2022-09-01

**Authors:** Yilun Deng, Simiao Wu, Junfeng Liu, Meng Liu, Lu Wang, JinCheng Wan, Shihong Zhang, Ming Liu

**Affiliations:** ^1^Department of Neurology, West China Hospital, Sichuan University, Chengdu, China; ^2^Department of Rehabilitation Medicine, West China Hospital, Sichuan University, Chengdu, China; ^3^Key Laboratory of Rehabilitation Medicine, West China Hospital, Sichuan University, Chengdu, China

**Keywords:** stress hyperglycemia ratio, glucose, cerebral edema, functional outcome, death

## Abstract

**Background and purpose:**

Absolute hyperglycemia at admission has been shown to be associated with the development of cerebral edema (CED) after acute cerebral infarction. Stress hyperglycemia is a more objective reflection of hyperglycemic state than absolute hyperglycemia. However, studies on the associations between stress hyperglycemia and CED are limited. We aimed to explore the associations of stress hyperglycemia, measured by stress hyperglycemia ratio (SHR), with the development of CED and poor functional outcome of acute cerebral infarction.

**Methods:**

Patients with acute middle artery cerebral infarction admitted to the Department of Neurology, West China Hospital of Sichuan University, within 24 h of symptom onset from January 2017 to March 2021 were included. Stress hyperglycemia was assessed by the SHR: admission fasting plasma glucose (FPG)/hemoglobin A1c (HbA1c). The primary outcome was the degree of CED evaluated on brain image. The secondary outcomes were moderate-to-severe CED, poor functional outcome (modified Rankin Scale score > 2), and death at 90 days. The associations between the SHR and outcomes were assessed with multivariate logistic regression analyses. We further compared the predictive value of the SHR, admission random plasma glucose (RPG), and admission FPG for outcomes in the training dataset and validation dataset.

**Results:**

638 patients were enrolled. Each 0.1-point increase in the SHR was independently associated with a 1.31-fold increased risk of a higher degree of CED [odds ratio (OR): 1.31 (95% confidence interval (CI): 1.20–1.42), *P* < 0.001]. The SHR was independently associated with moderate-to-severe CED [per 0.1-point increase: OR: 1.39 (95% CI: 1.24–1.57), *P* < 0.001], poor functional outcome [per 0.1-point increase: OR: 1.25 (95% CI: 1.12–1.40), *P* < 0.001], and death [per 0.1-point increase: OR: 1.13 (95% CI: 1.03–1.25), *P* < 0.05]. The predictive value of the SHR (as a continuous variable), exhibited by the area under the curve in receiver operating characteristic analysis, was higher than that of the RPG and FPG for moderate-to-severe CED and poor functional outcome (*P* < 0.05).

**Conclusion:**

The SHR is independently associated with the severity of CED, poor functional outcome, and death after acute cerebral infarction, and the SHR (as a continuous variable) has a better predictive value for moderate-to-severe CED and poor functional outcome than the RPG and FPG.

## Introduction

Cerebral edema (CED) is a pathophysiological process that occurs after acute cerebral infarction. Notably, the degree of CED often influences the prognosis of patients with acute cerebral infarction (Ferro et al., 2021). The mortality related to space-occupying infarcts by progressive CED within the first days after stroke onset can reach up to 80% (Berrouschot et al., 1998; Broocks et al., 2018). Recently, there has not been considerable progress in treatments to alleviate the development of CED. Therefore, identifying the related risk factors associated with progressive CED is important because they could guide early intervention.

Hyperglycemia is frequently observed in acute cerebral infarction, and it was estimated that 39–83% of diabetic patients and 8–63% of non-diabetic patients have elevated plasma glucose levels at admission (Kruyt et al., 2010). Stress reactions in acute severe illness or preexisting abnormalities in glucose metabolism have been proposed to account for hyperglycemia (Kruyt et al., 2010). Pathophysiologically, hyperglycemia can contribute to the damage of the blood–brain barrier (BBB) in acute ischemic stroke, which can then lead to brain edema or hemorrhagic transformation (Huang et al., 2013; Spronk et al., 2021). The absolute elevation of plasma glucose levels was found to be associated with malignant CED after large hemispheric infarction in previous studies (Shimoyama et al., 2014; Ong et al., 2017). Different from absolute hyperglycemia, stress hyperglycemia is a relative elevation of blood plasma glucose levels adjusted for background glucose, which is a more objective reflection of acute hyperglycemic state (Dungan et al., 2009; Roberts et al., 2015; Chen et al., 2022). Stress hyperglycemia is often evaluated by the stress hyperglycemia ratio (SHR) (Su et al., 2017; Zhu et al., 2019). The SHR is often defined as plasma glucose divided by hemoglobin A1c (HbA1c) and represents real transient hyperglycemia controlled for background plasma glucose (Su et al., 2017; Zhu et al., 2019). Stress hyperglycemia has been found to be associated with stroke recurrence, hemorrhagic transformation, neurological deficits, and mortality after acute ischemic stroke in many previous studies (Pan et al., 2017; Zhu et al., 2019; Li et al., 2020; Yuan et al., 2021). In addition, the SHR was shown to be a better prognostic indicator than the random plasma glucose (RPG) and fasting plasma glucose (FPG) for poor functional outcome in patients with acute cerebral infarction (Chen et al., 2022). However, to our knowledge, the number of previous studies on the association of stress hyperglycemia with the development of CED and its prognosis after acute cerebral infarction is limited, and a comparison of the predictive values of plasma glucose and the SHR for worse CED has not been reported.

The aim of our study was to explore the association of the SHR with the development of CED and its prognosis in patients with acute cerebral infarction and to compare the predictive values of the SHR, admission RPG, and FPG for worse CED and poor functional outcome.

## Materials and methods

### Study participants

We analyzed data from a prospectively collected database between January 2017 and March 2021, the Chengdu Stroke Registry described previously in Liu’s research (Liu et al., 2019), in which the patients were consecutively recruited at the Department of Neurology, West China Hospital, Sichuan University. To assess the predictive value of the SHR for moderate-to-severe CED and poor functional outcome, data from 2017 to 2019 formed the training dataset and those from 2020 to 2021 formed the validation dataset. The inclusion criteria were as follows: (1) age ≥ 18 years, (2) admission within 24 h after symptom onset, and (3) involvement of the middle cerebral artery (MCA) territory of infarction, with or without the involvement of the adjacent territories. The exclusion criteria were as follows: (1) absence of the FPG data within 48 h after symptom onset or the HbA1c data during their hospital stay, (2) absence of the admission RPG data upon arrival at the hospital, (3) absence of brain imaging within 24–120 h after symptom onset, (4) venous blood samples for testing FPG or RPG that were drawn after performing brain imaging, (5) parenchymal hemorrhage (PH) type 2 occurring before or at the same time as the head imaging to evaluate the degree of CED —the definition of PH type 2 is hemorrhage > 30% of the infarct area according to the European Cooperative Acute Stroke Study criteria (Hacke et al., 1995), and (6) bilateral cerebral infarction. This study was approved by the Biomedical Research Ethics Committee of West China Hospital, Sichuan University.

### Data collection

We collected information on demographics; time from onset; history of diabetes mellitus and other vascular risk factors; presence of symptomatic occlusion of major cerebral arteries relevant to acute ischemic lesions on computed tomography angiography (carotid artery or [middle cerebral artery (MCA)] M1–M2), magnetic resonance angiography or digital subtraction angiography; admission RPG, FPG, and other laboratory tests (if FPG was tested more than once during hospitalization, we chose the earliest one); HbA1c; and key treatments during hospitalization (acute endovascular treatment, intravenous thrombolysis, antihypertensive therapy, insulin, oral hypoglycemic agents, antiplatelet therapy, statin). Stroke severity was evaluated with the National Institutes of Health Stroke Scale (NIHSS) score on admission by well-trained neurologists. Stroke etiology was classified based on the Trial of ORG 10172 in Acute Stroke Treatment (TOAST) criteria (Adams et al., 1993). Diabetes mellitus was defined as physician-diagnosed diabetes mellitus. For patients who received acute endovascular treatment, successful [modified thrombolysis in cerebral infarction (mTICI) 2b–3] or unsuccessful reperfusion was also recorded (Dargazanli et al., 2018).

### Assessment of stress hyperglycemia

The SHR was calculated to assess stress hyperglycemia according to the following formula: FPG (mmol/L)/HbA1c (%) (Zhu et al., 2019). Fasting venous blood samples were drawn to measure FPG within 48 h after symptom onset during the morning hours (range: 04:00–12:00) after an overnight fast. HbA1c was measured during hospitalization. FPG was tested by an enzymatic method, and HbA1c was measured by high-performance liquid chromatography analysis in the Department of Laboratory Medicine, West China Hospital.

### Outcome measurements

The primary outcome was the CED grade on brain CT or brain MRI at 24–120 h after onset. The CED grade was classified according to the Safe Implementation of Thrombolysis in Stroke—Monitoring Study (SITS-MOST) protocol: CED-0: no CED; CED-1: focal brain swelling ≤ 1/3 of the hemisphere; CED-2: focal brain swelling > 1/3 of the hemisphere; and CED-3: brain swelling with midline shift (Wahlgren et al., 2007; Strbian et al., 2013). In patients who underwent brain imaging more than once, the most severe CED was chosen for assessment. Two well-trained neurologists reviewed the brain images. If there was disagreement, a decision was made with the help of a third neurologist. The secondary outcomes were moderate-to-severe CED (CED-2–3), 90-day poor functional outcome [modified Rankin Scale (mRS) score > 2 within 90 days after stroke], and 90-day death (death within 90 days regardless of causes). All included patients were followed up by telephone interviews 90 days after stroke.

### Statistical analysis

The associations between the SHR and CED grades were assessed using ordinal logistic regression after verification of the proportional odds assumption across all CED degrees. The associations of the SHR with moderate-to-severe CED, poor functional outcome, and death at 90 days were assessed using a binary logistic regression model. The variables with *P* < 0.05 in univariate analysis that changed the odds ratio of the SHR by at least 10 percent in a multivariate logistic regression model were included in the model (Kernan et al., 2000). Some vital clinical variables that might be associated with outcomes were also included in the multivariate logistic regression model. Subgroup analysis was preset to validate the robustness of the results. The following modifiers were included in the subgroup analysis: age (≥ 65 vs. < 65 years), baseline NIHSS score (≥15 vs. <15), diabetes mellitus, acute endovascular treatment, and intravenous thrombolysis. Receiver operator characteristic (ROC) curves were used to calculate the predictive values of SHR, FPG, and RPG as both continuous and binary variables for predicting moderate-to-severe CED, poor functional outcome, and death at 90 days in the training cohort and validation cohort. As a binary variable, FPG was dichotomized to ≥ 7 mmol/L and < 7 mmol/L and RPG was dichotomized to ≥ 10 mmol/L and < 10 mmol/L based on previous studies (Zhang et al., 2021; Mac Grory et al., 2022). For the SHR, the ROC curve in the training cohort was applied to identify an optimized cutoff value (the maximum value of the Youden index) of the SHR to predict the onset of moderate-to-severe CED. The predictive value of that cutoff value for outcomes was assessed in both the training cohort and the validation cohort. The DeLong test package in MedCalc was used to compare the area under the curve (AUC) values of the SHR, FPG, and RPG. All statistical analyses were performed using R version 4.0.4 (R Foundation for Statistical Computing, Vienna, Austria), MedCalc 20.027 (MedCalc, Belgium), and SPSS version 25.0 (IBM Corp., Armonk, NY, United States). A two-tailed *P* < 0.05 was considered statistically significant.

## Results

### Baseline characteristics

**FIGURE 1:**
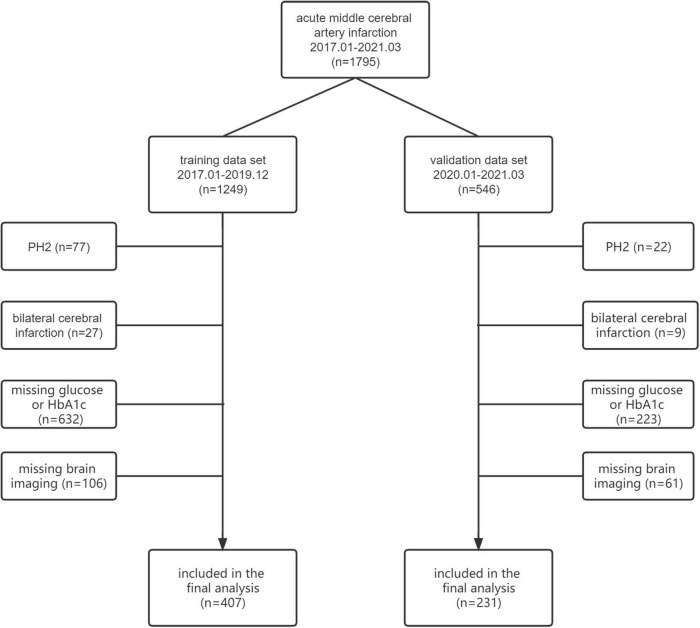
Flowchart of the screening of patients. PH, Parenchymal hemorrhage; HbA1c, hemoglobin A1c.

From January 2017 to March 2021, 1795 patients who were diagnosed with acute middle cerebral artery infarction within 24 h after stroke onset were screened. Among them, 638 patients met the inclusion criteria, but not the exclusion criteria, for the final analysis (training dataset: *n* = 407; validation dataset: *n* = 231) (Figure 1). Baseline characteristics were well balanced between the included and excluded patients except for hyperlipidemia, previous ischemic stroke/TIA, and valvular heart disease (Supplementary Table 1). The baseline characteristics of the training and validation datasets are shown in Table 1. In the training dataset, the median [interquartile range (IQR)] age was 70 (61–79) years, 235 patients (57.7%) were male, and the median (IQR) NIHSS score was 10 (3–16). In the validation dataset, the median (IQR) age was 70 (62–78) years, 143 patients (61.9%) were male, and the median (IQR) NIHSS score was 10 (4–15). Compared with patients in the training dataset, those in the validation dataset were more likely to have hypertension (*P* = 0.019), hyperlipidemia (*P* = 0.001), diabetes mellitus (*P* = 0.029), large artery atherosclerosis and small artery occlusion as TOAST subtypes (*P* = 0.022), large artery occlusion (carotid artery or MCA M1-M2) (*P* = 0.004), statin treatment (*P* = 0.046), and endovascular treatment (*P* < 0.001). Patients in the validation dataset had a longer onset to admission time (*P* = 0.004) and higher FPG (*P* = 0.002) and SHR (*P* = 0.004) values. Fewer patients in the validation dataset had a history of smoking (*P* = 0.018) and previous stroke/transient ischemic stroke (TIA) (*P* = 0.009). In the training dataset, blood samples for testing FPG were collected at a median (IQR) time of 23.4 (16.8–41.6) h from onset, and the median (IQR) time from onset to brain imaging to assess the degree of CED was 56.1 (41.8–76.9) h. In the validation dataset, blood samples for testing FPG were collected at a median (IQR) time of 25.0 (17.8–41.9) h from onset, and the median time from onset to brain imaging to assess the degree of CED was 57.9 (45.0–78.1) h. The median (IQR) SHR value was 1.00 (0.88–1.16) in the training dataset and 1.06 (0.89–1.30) in the validation dataset. There were 20 patients lost to follow-up at 90 days in the training dataset (4.9%) and 21 patients in the validation dataset (9.1%).

**TABLE 1:** Baseline characteristics of the training dataset and validation dataset.

Variables	Training dataset (*n* = 407)	Validation dataset (*n* = 231)	*P*-value
Age (year), median (IQR)	70 (61–79)	70 (62–78)	0.959
Male, *n* (%)	235 (57.7)	143 (61.9)	0.303
Onset to admission time (hour), median (IQR)	5 (3–24)	9 (4–24)	0.004
Vascular risk factors, *n* (%)			
Hypertension, *n* (%)	220 (54.1)	147 (63.6)	0.019
Hyperlipidemia, *n* (%)	19 (4.7)	27 (11.7)	0.001
Diabetes mellitus, *n* (%)	90 (22.1)	69 (29.9)	0.029
Atrial fibrillation/Atrial flutter, *n* (%)	146 (35.9)	73 (31.6)	0.275
Valvular heart disease, *n* (%)	43 (10.6)	16 (6.9)	0.127
Previous ischemic stroke/TIA, *n* (%)	49 (12.0)	13 (5.6)	0.009
Smoking, *n* (%)	131 (32.2)	54 (23.4)	0.018
Alcohol consumption, *n* (%)	63 (15.5)	50 (21.6)	0.05
Baseline NIHSS, median (IQR)	10 (3–16)	10 (4–15)	0.679
TOAST subtypes, *n* (%)			0.022
Large artery atherosclerosis	124 (30.5)	91 (39.4)	
Small artery occlusion	63 (15.5)	47 (20.3)	
Cardioembolism	133 (32.7)	57 (24.7)	
Other etiology	6 (1.5)	2 (0.9)	
Undetermined	81 (19.9)	34 (14.7)	
Occlusion site, *n* (%)			0.004
Carotid occlusion	75 (18.4)	60 (26.0)	
MCA occlusion (M1–M2)	130 (31.9)	87 (37.7)	
No record or other	202 (49.6)	84 (36.4)	
Treatment during hospitalization, *n* (%)			
Endovascular treatment	68 (16.7)	86 (37.2)	<0.001
Intravenous thrombolysis	57 (14.0)	41 (17.7)	0.211
Antihypertensive therapy	151 (37.1)	95 (41.1)	0.315
Insulin	54 (13.3)	34 (14.7)	0.61
Oral hypoglycemic agents	54 (13.3)	39 (16.9)	0.214
Antiplatelet	357 (87.7)	205 (88.7)	0.7
Statin	350 (86.0)	211 (91.3)	0.046
mTICI 2b-3*, *n* (%)	60 (88.2)	79 (91.9)	0.451
RPG (mmol/l), median (IQR)	7.36 (6.27–9.00)	7.26 (6.34–9.13)	0.488
FPG (mmol/l), median (IQR)	5.94 (5.19–7.32)	6.48 (5.28–8.34)	0.002
HbA1c (%), median (IQR)	6.00 (5.60–6.40)	5.90 (5.60–6.60)	0.557
SHR, median (IQR)	1.00 (0.88–1.16)	1.06 (0.89–1.30)	0.004

IQR, interquartile range; TIA, transient ischemic attack; NIHSS, the National Institutes of Health Stroke Scale; TOAST, Trial of Org 10172 in Acute Stroke Treatment; MCA, middle cerebral artery; mTICI, modified Thrombolysis in Cerebral Infarction; RPG, random plasma glucose; FPG, fasting plasma glucose; HbA1c, hemoglobin A1c; SHR, stress hyperglycemia ratio. *The proportion of the patients receiving endovascular treatment.

### Association between the stress hyperglycemia ratio and cerebral edema

In univariate analysis, the SHR; sex; atrial fibrillation/atrial flutter; onset to admission time; baseline NIHSS score; diabetes mellitus; large artery atherosclerosis, cardioembolism, and other etiologies of the TOAST classification; large artery occlusion [carotid occlusion or MCA occlusion (M1–M2)]; endovascular treatment; intravenous thrombolysis; oral hypoglycemic agents; and antiplatelet therapy were found to be associated with a worse CED grade (Table 2). In ordinal logistic regression (Table 2), the correlation with the SHR was significant [per 0.1-unit increase: odds ratio (OR): 1.31, 95% confidence interval (CI): 1.20–1.42, *P* < 0.001] after adjusting for potential covariates from the univariate analysis and some variables of clinical significance, such as age and RPG.

**TABLE 2:** Associated factors for the development of CED on training dataset.

Variables	Univariate analysis, OR (95%CI)	*P*-value	Multivariate analysis, OR (95%CI)	*P*-value
Age	1.01 (1.00–1.03)	0.063	1.01 (0.99–1.02)	0.44
Male	0.65 (0.45–0.94)	0.023		
Hypertension	0.97 (0.67–1.39)	0.861		
Hyperlipidemia	0.99 (0.42–2.32)	0.976		
Atrial fibrillation/Atrial flutter	2.70 (1.84–3.97)	<0.001		
Previous ischemic stroke/TIA	0.66 (0.37–1.16)	0.146		
Valvular heart disease	1.00 (0.56–1.79)	0.992		
Smoking	0.70 (0.48–1.04)	0.075		
Alcohol consumption	0.67 (0.41–1.12)	0.128		
Diabetes mellitus	0.51 (0.32–0.80)	0.003	0.36 (0.19–0.69)	0.002
Onset to admission time	0.96 (0.94–0.98)	<0.001		
Baseline NIHSS	1.16 (1.13–1.19)	<0.001	1.10 (1.06–1.13)	<0.001
TOAST classification				
Large artery atherosclerosis	13.95 (6.14–31.74)	<0.001	3.4 (1.36–8.49)	0.009
Cardioembolism	17.31 (7.63–39.28)	<0.001	3.19 (1.26–8.03)	0.014
Others	12.72 (5.45–29.71)	<0.001	5.44 (2.18–13.54)	<0.001
Small-artery occlusion	Reference		Reference	
Occlusion site				
Carotid occlusion	14.68 (8.40–25.66)	<0.001	6.85 (3.63–12.94)	<0.001
MCA occlusion (M1–M2)	5.89 (3.75–9.26)	<0.001	3.83 (2.19–6.69)	<0.001
No record or other	Reference		Reference	
Endovascular treatment	2.28 (1.41–3.70)	0.001	0.41 (0.22–0.77)	0.005
Intravenous thrombolysis	1.70 (1.02–2.85)	0.044	1.19 (0.67–2.11)	0.56
Antihypertensive therapy	0.83 (0.57–1.21)	0.332		
Insulin	0.81 (0.47–1.38)	0.43		
Oral hypoglycemic agents	0.46 (0.26–0.80)	0.006		
Antiplatelet	0.40 (0.23–0.70)	0.001		
Statin	0.73 (0.43–1.22)	0.224		
mTICI (2b/3)	0.29 (0.07–1.19)	0.087		
RPG	0.98 (0.93–1.04)	0.535	1.03 (0.95–1.12)	0.52
SHR (per 0.1-point increases)	1.33 (1.24–1.43)	0.001	1.31 (1.20–1.42)	<0.001

OR, odds ratio; CI, confidence interval; TIA, transient ischemic attack; NIHSS, the National Institutes of Health Stroke Scale; TOAST, Trial of Org 10172 in Acute Stroke Treatment; MCA, middle cerebral artery; mTICI, modified Thrombolysis in Cerebral Infarction; RPG, random plasma glucose; SHR, stress hyperglycemia ratio.

**FIGURE 2:**
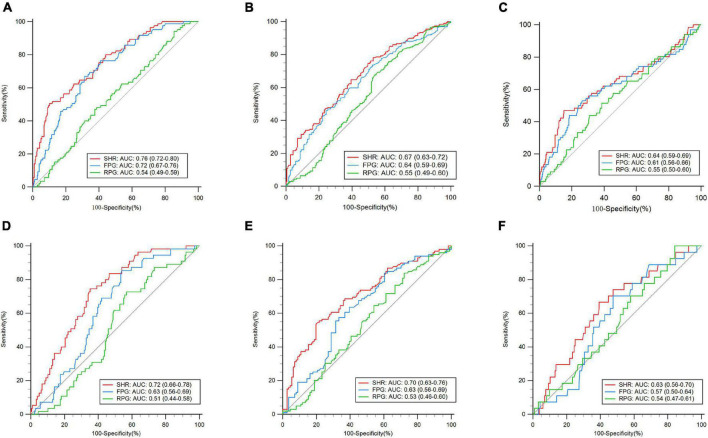
Receiver operating characteristic curve (ROC) analyses of the stress hyperglycemia ratio (SHR), admission fasting plasma glucose (FPG), and admission random plasma glucose (RPG) for the prediction of outcomes. Training dataset: **(A)** moderate-to-severe cerebral edema (CED), **(B)** 90-day poor functional outcome, and **(C)** 90-day death. Validation dataset: **(D)** moderate-to-severe CED, **(E)** 90-day poor functional outcome, and **(F)** 90-day death.

Eighty-five patients in the training dataset developed moderate-to-severe CED (20.9%). A ROC curve showed that the optimized cutoff value of the SHR to predict moderate-to-severe CED was 1.25 (Figure 2A). The SHR was dichotomized into groups of high (≥ 1.25) and low (< 1.25) SHR. In the training dataset, a higher SHR (≥ 1.25) remained independently associated with moderate-to-severe CED in multivariate logistic regression (OR: 6.87, 95% CI: 3.46–13.63, *P* < 0.001) (Supplementary Table 2).

### Association between the stress hyperglycemia ratio and 90-day poor functional outcome

Two hundred and two patients in the training dataset had poor functional outcome (52.2%). In univariate analysis, the SHR; age; sex; atrial fibrillation/atrial flutter; previous ischemic stroke/TIA; baseline NIHSS score; large artery atherosclerosis, cardioembolism, and other etiologies of the TOAST classification; and large artery occlusion [carotid occlusion or MCA occlusion (M1–M2)] were associated with 90-day poor functional outcome (Table 3). The SHR was independently associated with the 90-day poor functional outcome after adjustment for potential confounders in model 1 (per 0.1-point increases: OR: 1.25, 95% CI: 1.12–1.40, *P* < 0.001) (Table 3). In model 2, compared with patients with a lower SHR, those with a higher SHR (≥ 1.25) had a 3.73-fold higher risk of poor functional outcome at 90 days (OR: 3.73, 95% CI: 1.74–7.97, *P* < 0.001) (Table 3).

**TABLE 3:** Predictive factors for the development of 90-day poor outcome (mRS > 2) on training dataset.

Variables	Univariate analysis, OR (95%CI)	*P*-value	Multivariate analysis OR (95%CI), model 1	*P*-value	Multivariate analysis OR (95%CI), model 2	*P*-value
Age	1.04 (1.02–1.06)	<0.001	1.04 (1.02, 1.06)	<0.001	1.04 (1.02, 1.06)	<0.001
Male	0.58 (0.39–0.87)	0.009				
Hypertension	1.18 (0.79–1.76)	0.43				
Hyperlipidemia	1.46 (0.56–3.86)	0.441				
Atrial fibrillation/Atrial flutter	2.35 (1.53–3.60)	<0.001				
Previous ischemic stroke/TIA	1.99 (1.05–3.76)	0.034	2.37 (1.08, 5.17)	0.031	2.30 (1.07, 4.94)	0.033
Valvular heart disease	1.19 (0.62–2.29)	0.597				
Smoking	0.90 (0.59–1.39)	0.636				
Alcohol consumption	0.59 (0.33–1.05)	0.074				
Diabetes mellitus	0.79 (0.49–1.28)	0.342	0.54 (0.25, 1.13)	0.102	0.64 (0.31, 1.31)	0.221
Onset to admission time	1.00 (0.98–1.02)	0.979				
Baseline NIHSS	1.14 (1.10–1.18)	<0.001	1.14 (1.09, 1.19)	<0.001	1.14 (1.10, 1.19)	<0.001
TOAST classification						
Large artery atherosclerosis	3.44 (1.71–6.89)	0.001				
Cardioembolism	4.18 (2.10–8.34)	<0.001				
Others	2.54 (1.22–5.29)	0.013				
Small-artery occlusion	Reference		Reference		Reference	
Occlusion site						
Carotid occlusion	5.11 (2.73–9.58)	<0.001	3.10 (1.41, 6.81)	0.005	3.05 (1.40, 6.64)	0.005
MCA occlusion (M1–M2)	1.76 (1.12–2.78)	0.014	1.13 (0.61, 2.09)	0.695	1.14 (0.62, 2.09)	0.676
No record or other	Reference		Reference		Reference	
Endovascular treatment	1.34 (0.79–2.29)	0.28	0.45 (0.21, 0.94)	0.035	0.54 (0.26, 1.13)	0.101
Intravenous thrombolysis	0.67 (0.38–1.19)	0.172	0.41 (0.20, 0.82)	0.012	0.40 (0.20, 0.80)	0.01
Antihypertensive therapy	0.95 (0.63–1.44)	0.807				
Insulin	1.19 (0.66–2.17)	0.564				
Oral hypoglycemic agents	0.76 (0.42–1.36)	0.349				
Antiplatelet	0.54 (0.29–1.03)	0.062				
Statin	0.63 (0.35–1.14)	0.125				
mTICI (2b/3)	0.42 (0.079–2.27)	0.316				
RPG	1.00 (0.94–1.07)	0.913	1.03 (0.94, 1.13)	0.5	1.04 (0.95, 1.13)	0.368
SHR (per 0.1-point increases)	1.32 (1.20–1.45)	<0.001	1.25 (1.12, 1.40)	<0.001		
SHR (≥ 1.25)	5.46 (2.88–10.35)	<0.001			3.73 (1.74, 7.97)	0.001

OR, odds ratio; CI, confidence interval; TIA, transient ischemic attack; NIHSS, National Institutes of Health Stroke Scale; TOAST, Trial of Org 10172 in Acute Stroke Treatment; MCA, middle cerebral artery; mTICI, modified Thrombolysis in Cerebral Infarction; RPG: random plasma glucose; SHR, stress hyperglycemia ratio.

### Association between the stress hyperglycemia ratio and 90-day death

Sixty-six patients in the training dataset died (17.1%). In univariate analysis, the SHR; age; baseline NIHSS score; large artery atherosclerosis and cardioembolism of the TOAST classification; carotid occlusion; antiplatelet use; and statin use were associated with 90-day death (Supplementary Table 3). The SHR was independently associated with 90-day death with adjustment for potential confounders in model 1 (per 0.1-point increases: OR: 1.13, 95% CI: 1.03–1.25, *P* = 0.01). In model 2, compared with patients with a lower SHR, those with a higher SHR (≥ 1.25) had a 2.79-fold higher risk of death at 90 days (OR: 2.79, 95% CI: 1.42–5.49, *P* = 0.003).

### Subgroup analysis

Subgroup analysis was performed by age (≥ 65 vs. < 65 years), baseline NIHSS score (≥ 15 vs. < 15), diabetes mellitus, endovascular therapy, and intravenous thrombolysis (Supplementary Figures 1–3). There was a stronger association of a higher SHR with 90-day poor functional outcome (OR: 2.34 vs. 23.07; *P* = 0.027) in patients who received acute endovascular therapy compared with those who did not receive acute endovascular therapy (Supplementary Figure 2). In other subgroup analyses, no significant interaction between the SHR and stratified variables was observed (*P* > 0.05).

### Receiver operator characteristic analysis

ROC analysis was applied to assess the performance of the SHR in predicting moderate-to-severe CED, 90-day poor functional outcome, and 90-day death (Figure 2). In the training dataset, compared with the RPG and FPG, the SHR had a significantly higher AUC value in the prediction of moderate-to-severe CED (AUC: 0.76, 95% CI: 0.72–0.80, *P* < 0.001) (*P* < 0.01). For the 90-day poor functional outcome, the predictive value of the SHR, as indicated by the AUC, was 0.67 (95% CI: 0.63–0.72, *P* < 0.01), which was significantly higher than that of the RPG (*P* < 0.01) and FPG (*P* = 0.049). For 90-day death, the predictive value of the SHR exhibited by the AUC was 0.64 (95% CI: 0.59–0.69, *P* = 0.0011), which was not significantly different from that of RPG or FPG (*P* > 0.05). In the validation dataset, for moderate-to-severe CED, the predictive value of the SHR (AUC: 0.72, 95% CI: 0.66–0.78, *P* < 0.001) was higher than that of the RPG and FPG (*P* < 0.01). For the 90-day poor functional outcome, the predictive value of the SHR (AUC: 0.70, 95% CI: 0.63–0.76, *P* < 0.001) was also higher than that of the RPG and FPG (*P* < 0.05). In the prediction of 90-day death, the SHR had a significantly higher AUC (AUC: 0.63, 95% CI: 0.56–0.70, *P* = 0.017) than the FPG (*p* < 0.01), while there was no significant difference compared with the RPG (*P* = 0.31).

**FIGURE 3:**
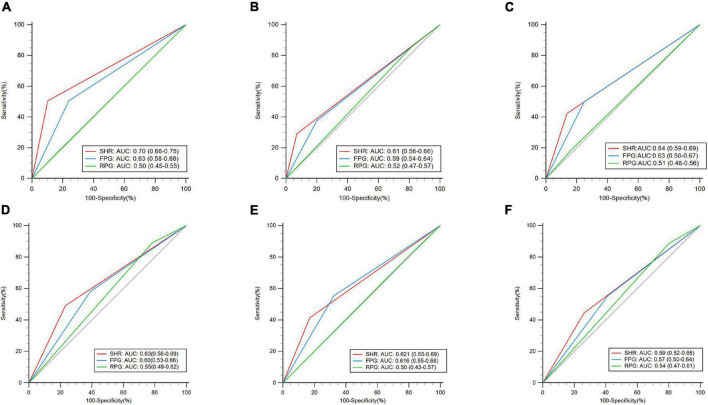
Receiver operating characteristic curve (ROC) analyses of the stress hyperglycemia ratio (SHR), admission fasting plasma glucose (FPG), and admission random plasma glucose (RPG) as binary variables for the prediction of outcomes. Training dataset: **(A)** moderate-to-severe cerebral edema (CED), **(B)** 90-day poor functional outcome, and **(C)** 90-day death. Validation dataset: **(D)** moderate-to-severe CED, **(E)** 90-day poor functional outcome, and **(F)** 90-day death.

As binary variables, for moderate-to-severe CED, in the training dataset, the predictive value of the SHR (≥ 1.25 vs. < 1.25) (AUC: 0.7, 95% CI: 0.66–0.75, *P* < 0.01) was higher than that of the RPG (≥ 10 vs. < 10) and FPG (≥ 7 vs. < 7) (*P* < 0.01) (Figure 3). For the 90-day poor functional outcome, the SHR (AUC: 0.61, 95% CI: 0.56–0.66, *P* < 0.01) had a significantly higher AUC value than the RPG (*P* < 0.01), but there was no significant difference between the AUCs of the SHR and FPG (*P* = 0.23). For 90-day death, the predictive value of the SHR (AUC: 0.64, 95% CI: 0.59–0.69) was higher than that of the RPG (*P* < 0.01), while there was no significant difference in the AUC between the SHR and FPG (*P* = 0.41). In the validation dataset, for moderate-to-severe CED and 90-day death, there was no significant difference in AUCs between the SHR and FPG, or RPG (*P* > 0.05). For the 90-day poor functional outcome, the predictive value of the SHR (AUC: 0.62, 95% CI: 0.55–0.69, *P* < 0.01) was higher than that of the RPG (*P* = 0.0069), while there was no significant difference in the AUCs between the SHR and FPG (*P* = 0.82). The sensitivity, specificity, positive predictive value, negative predictive value, and accuracy of the SHR in the prediction of outcomes are presented in Supplementary Table 4.

## Discussion

The main finding of our study is that the SHR is independently associated with the development of CED, poor functional outcome, and death at 90 days. Moreover, the SHR (as a continuous variable) has a better predictive value for moderate-to-severe CED and 90-day poor functional outcome than the RPG and FPG.

To our knowledge, few studies have focused on the SHR with CED (Cannarsa et al., 2022) and there have been no studies comparing the SHR with the FPG or RPG in the prediction of CED. Previous literature has mostly focused on the prognosis of mild stroke or a cohort of different acute ischemic stroke types (Pan et al., 2017; Tziomalos et al., 2017; Merlino et al., 2020; Yuan et al., 2021). In those studies, stress hyperglycemia was shown to be associated with a high risk of stroke recurrence, hemorrhagic transformation, or a worse long-term outcome. Moreover, stress hyperglycemia was found to be associated with acute cerebrovascular events because it might aggravate oxidative stress and endothelial dysfunction (Pan et al., 2017).

Stress hyperglycemia, measured by the SHR, which is defined as FPG/HbA1c, is a reliable reflection of a transient increase in blood glucose levels adjusting for the background blood glucose level (Su et al., 2017; Zhu et al., 2019). Compared with the chronic status of hyperglycemia, acute hyperglycemia is associated with greater oxidative stress, an increase in inflammatory factor levels, and neurohormonal derangements such as excessive elevations of glucagon, epinephrine, cortisol, tumor necrosis factor-α (TNF-α), and interleukin-1 levels (Dungan et al., 2009). The acute elevation of blood glucose levels in turn exacerbates these inflammatory factors, which might form a vicious cycle (Dungan et al., 2009). Hyperglycemia plays a critical role in the destruction of BBB integrity mediated by inflammatory factors and oxidative stress (Spronk et al., 2021). In addition, acute glucose fluctuation is harmful to the intact endothelium and promotes a stronger oxidative stress response (Dungan et al., 2009). Hyperglycemia was shown to aggravate CED by disruption of the BBB in animal studies (Huang et al., 2013; Yuen et al., 2019). Our findings provide further evidence for the association between the SHR and the severity of CED after stroke.

For moderate-to-severe CED, 90-day poor functional outcome, and 90-day death, there was no significant interaction between the admission NIHSS score and the higher SHR (≥ 1.25) with respect to the outcomes. While a previous study found that stress hyperglycemia only reflected stroke severity rather than having a direct association with adverse outcomes, the researchers found that patients with stress hyperglycemia had more severe stroke; however, when including the NIHSS score, stress hyperglycemia was not significantly associated with adverse outcomes (Tziomalos et al., 2017). Thus, the researchers believed that the SHR was a marker of the stress response mediated by cortisol levels, which was not directly associated with adverse outcomes (Tziomalos et al., 2017). In our study, when the NIHSS score was included in the multivariate model, the SHR was still independently associated with the outcomes. Our findings suggested that the SHR was directly associated with moderate-to-severe CED and poor functional outcome and was more than a marker of the stress response. Similar to our findings, previous literature suggested that the acute elevation of glucose levels in acute illness might promote a stronger inflammatory response, which can contribute to the disruption of the BBB (Spronk et al., 2021). Disruption of the BBB can lead to the development of CED or hemorrhagic transformation.

The definition of the stress hyperglycemia has varied in previous studies, and many studies focused on a stroke population without a history of diabetes mellitus, which might be due to the lack of a consensus definition of a cutoff value for stress hyperglycemia for patients with preexisting diabetes mellitus (Dungan et al., 2009; Yoon et al., 2016; Tziomalos et al., 2017; Zhu et al., 2019). However, considering patients with diagnosed diabetes mellitus is necessary because stress hyperglycemia can also occur in patients with diabetes mellitus. In our study, multivariate logistic regression showed that the SHR was independently positively associated with moderate-to-severe CED, 90-day poor functional outcome, and 90-day death, and further subgroup analysis showed no significant interaction between diabetes mellitus status and the higher SHR (≥ 1.25) for the outcomes. Our result was similar to previous literature which demonstrated that elevated plasma glucose levels were prominently associated with poor outcomes regardless of a history of diabetes mellitus in acute ischemic stroke or acute myocardial infarction (Ishihara et al., 2007; Tsivgoulis et al., 2019).

For the 90-day poor functional outcome, we found that the positive effect of the SHR was more significant in patients who received acute endovascular therapy than in those who did not, with interactions between the SHR level and endovascular treatment status. Cannarsa suggested that the SHR was associated with malignant CED, intracranial hemorrhage, and poor functional outcome after mechanical thrombectomy, and the blood sample for testing plasma glucose was collected before mechanical thrombectomy (Cannarsa et al., 2022). In our study, the blood sample of testing FPG, which was used to evaluate the SHR, was obtained after acute endovascular treatment. Merlino showed stress hyperglycemia was associated with 90-day poor outcomes in patients undergoing mechanical thrombectomy, and the time of testing FPG was after mechanical thrombectomy as well (Merlino et al., 2021). Thus, it is necessary to be concerned about plasma glucose levels after acute endovascular treatment. Similar to our findings, a previous meta-analysis showed that there was a significant interaction between plasma glucose levels and endovascular treatment status (Chamorro et al., 2019). It was proposed that the redox-mediated harmful effects of glucose are common in endovascular treatment when successful reperfusion is achieved (Chamorro et al., 2019).

Admission plasma glucose has been demonstrated to be a risk factor associated with CED after acute ischemic stroke in previous studies (Shimoyama et al., 2014; Cheng et al., 2020; Dowlati et al., 2021). However, our findings showed that the RPG was not associated with this outcome, while the SHR was independently associated with a higher degree of CED, 90-day poor functional outcome, and 90-day death, and the predictive value of the SHR (as a continuous variable) for moderate-to-severe CED and 90-day poor functional outcome, indicated by the AUC, was better than that of the RPG or FPG. Previous literature showed that the SHR was more strongly associated with outcomes than glucose levels: A previous study showed that stress hyperglycemia was associated with poor outcomes of acute cerebral infarction, such as stroke exacerbation, inpatient mortality, or functional deficits at discharge, while glucose levels were not (Roberts et al., 2021). In another study, glucose levels were not associated with critical illness (in-hospital death or critical care) in multivariate logistic regression analysis, while the SHR maintained a significant association. The definition of the SHR in that article was glucose divided by estimated glucose derived from HbA1c, which is similar to the concept of the SHR in our study (Roberts et al., 2015). Similarly, the SHR, rather than glucose, was considered to be a risk factor associated with in-hospital mortality in patients with acute myocardial infarction (Chen et al., 2021). Su’s study showed that the SHR had an AUC value of 0.67, while glucose had an AUC value of only 0.52 in the prediction of 90-day all-cause mortality in acute illness (Su et al., 2017). It was proposed that the elevation of absolute glucose levels did not accurately reflect the real stress hyperglycemia state during acute illness without considering the impact of background plasma glucose. To the best of our knowledge, there is still a lack of research comparing the predictive value of admission plasma glucose with the SHR for CED. Previous predictive models of malignant CED, such as the Enhanced Detection of Edema in Malignant Anterior Circulation Stroke (EDEMA) score and DASH score, included admission plasma glucose as an item (Shimoyama et al., 2014; Ong et al., 2017). However, according to our study and previous literature, it was suggested to further compare the predictive power of the SHR and RPG in those models.

The baseline characteristics between the training dataset and the validation dataset were not totally similar. The main reason might be the development of acute endovascular treatment in our hospital in recent years, which led to the increasing number of patients with large cerebral artery occlusion and large artery atherosclerosis as TOAST subtypes. Notably, our results showed the SHR had the highest AUC value for outcomes in both the training dataset and the validation dataset, which reflected the transportability of our model.

The strength of this study is that the comparison of the predictive values of the RPG, FPG, and SHR for worse CED in acute cerebral infarction has not been explored before, to our knowledge. However, there were some limitations of our study: First, patients were excluded due to the lack of RPG, FPG, HbA1c, and brain imaging data, which might cause selection bias, and we compared the baseline information between the excluded and included patients to compensate for this. Second, our study was a single-center study and the results of our study should be validated by multicenter studies in future. Third, our studies only focused on patients with acute middle cerebral infarction with or without the involvement of the adjacent territories, not for other types.

## Conclusion

In our study, the SHR, measured by the FPG/HbA1c ratio, is independently associated with CED, poor functional outcome, and death after acute cerebral infarction. The SHR (as a continuous variable) appears to have a better predictive value for moderate-to-severe CED and poor functional outcome than the RPG and FPG. In future studies, exploring whether the predictive value of the model consisting of traditional risk factors for the development of CED will be improved by adding the SHR is significant.

## Data availability statement

The raw data supporting the conclusions of this article will be made available by the authors, without undue reservation.

## Ethics statement

The studies involving human participants were reviewed and approved by the Biomedical Research Ethics Committee of West China Hospital, Sichuan University. Written informed consent for participation was not required for this study in accordance with the national legislation and the institutional requirements.

## Author contributions

YD contributed to the study design and data analysis and wrote the manuscript. MiL and SZ contributed to the study design and data analysis and edited the manuscript. SW, JL, and JW assembled the collected data. MeL and LW contributed to the study design and discussion. All authors contributed to the article and approved the submitted version.
