# Supplementary material for: The stress hyperglycemia ratio is associated with the development of cerebral edema and poor functional outcome in patients with acute cerebral infarction
Source: Front Aging Neurosci. 2022 Sep 1;14:936862. doi: 10.3389/fnagi.2022.936862 (PMC9474997; doi:10.3389/fnagi.2022.936862)
Supplement: Supplementary file 1 [file Presentation_1.pdf]

Supplementary Material

1. Supplementary Figures and Tables

Supplementary Table 1. Baseline characteristics of patients included and excluded.

Variables	Included(n=638)	Excluded for missing glucose, HbA1c or brain imaging (n=1022)	*P* value
Age(year), median (IQR)	70(61-79)	70(59-79)	0.69
Male, n (%)	378(59.2)	575(56.3)	0.231
Hypertension, n (%)	367(57.5)	593(58.0)	0.841
Hyperlipidemia, n (%)	46(7.2)	116(11.4)	0.006
Diabetes mellitus, n (%)	159(24.9)	238(23.3)	0.448
Atrial fibrillation/Atrial flutter, n (%)	219(34.3)	380(37.2)	0.239
Previous ischemic stroke/TIA, n (%)	63(9.9)	145(14.2)	0.01
Valvular heart disease, n (%)	59(9.2)	130(12.7)	0.03
Smoking, n (%)	225(35.3)	351(34.3)	0.701
Alcohol consumption, n (%)	177(27.7)	272(26.6)	0.615
Baseline NIHSS, median (IQR)	10(3-15)	9(3-15)	0.06

HbA1c, hemoglobin A1c; IQR. interquartile range; TIA. transient ischemic attack; NIHSS: National Institutes of Health Stroke Scale

Supplementary Table 2. Predictive factors for the development of moderate-to-severe CED on training dataset

Variables	Univariate analysis OR (95%CI)	*P* value	Multivariate analysis OR (95%CI), model 1	*P* value	Multivariate analysis OR (95%CI), model 2	*P* value
Age	1.01(0.99-1.03)	0.274	1.00(0.98-1.03)	0.697	1.01(0.98-1.03)	0.653
Male	0.83(0.51-1.34)	0.448				
Hypertension	1.13(0.70-1.83)	0.615				
Hyperlipidemia	1.38(0.48-3.93)	0.552				
Atrial fibrillation/Atrial flutter	2.62(1.61-4.26)	<0.001				
Previous stroke/TIA	0.84(0.39-1.80)	0.644				
Valvular heart disease	0.85(0.38-1.91)	0.698				
Diabetes mellitus	0.64(0.34-1.20)	0.161	0.27(0.09-0.79)	0.016	0.44(0.17-1.19)	0.108
Smoking	0.79(0.47-1.34)	0.381				
Alcohol consumption	0.68(0.33-1.39)	0.29				
Onset to admission time	0.95(0.92-0.98)	0.002				
Baseline NIHSS(⩾15)	4.32(2.62-7.13)	<0.001	1.93(1.05-3.57)	0.036	2.23(1.21-4.11)	0.01
Occlusion site						
No record or other	Reference		Reference		Reference	
Carotid occlusion	27.71(11.96-64.20)	<0.001	18.44(7.28-46.70)	<0.001	16.45(6.57-41.20)	<0.001
MCA occlusion (M1-M2)	9.65(4.32-21.54)	<0.001	8.47(3.44-20.87)	<0.001	7.89(3.24-19.23)	<0.001
Endovascular treatment	2.10(1.18-3.73)	0.012	0.43(0.20-0.92)	0.03	0.58(0.28-1.21)	0.149
Intravenous thrombolysis	1.43(0.75-2.72)	0.279	1.27(0.57-2.79)	0.559	1.33(0.61-2.89)	0.479
Antihypertensive therapy	0.91(0.55-1.49)	0.698				
Insulin	1.24(0.63-2.43)	0.536				
Oral hypoglycemic agents	0.62(0.28-1.38)	0.242				
Antiplatelet	0.37(0.20-0.70)	0.002				
Statin	0.78(0.41-1.51)	0.462				
mTICI (2b/3)	0.24(0.05-1.10)	0.067				
RPG	1.00(0.93-1.08)	0.964	1.03(0.90-1.18)	0.633	1.04(0.92-1.18)	0.535
SHR (per 0.1-point increases)	1.40(1.27-1.54)	<0.001	1.39(1.24-1.57)	<0.001		
SHR(⩾1.25)	8.97(5.14-15.65)	<0.001			6.87(3.46-13.63)	<0.001

OR, odds ratio; CI, confidence interval; TIA. transient ischemic attack; NIHSS, the National Institutes of Health Stroke Scale; MCA, middle cerebral artery; mTICI. modified Thrombolysis in Cerebral Infarction; RPG: random plasma glucose; SHR. stress hyperglycemia ratio

Supplementary Table 3. Predictive factors for the development of 90-day death on training dataset

Variables	Univariate analysis OR (95%CI)	*P* value	Multivariate analysis OR (95%CI), model 1	*P* value	Multivariate analysis OR (95%CI), model 2	*P* value
Age	1.04(1.02-1.07)	0.001	1.03(1.01-1.06)	0.018	1.03(1.00-1.05)	0.02
Male	1.15(0.67-1.96)	0.62				
Hypertension	1.16(0.68-1.98)	0.585				
Hyperlipidemia	1.94(0.67-5.65)	0.223				
Atrial fibrillation/Atrial flutter	1.30(0.76-2.23)	0.337				
Previous ischemic stroke/TIA	1.33(0.63-2.83)	0.458	1.04(0.44-2.46)	0.92	1.08(0.46-1.53)	0.87
Valvular heart disease	1.20(0.53-2.74)	0.658				
Smoking	0.72(0.40-1.32)	0.291				
Alcohol consumption	0.78(0.35-1.75)	0.552				
Diabetes mellitus	1.27(0.69-2.34)	0.449	0.85(0.37-1.99)	0.71	0.97(0.43-2.22)	0.95
Onset to admission time	0.99(0.96-1.02)	0.387				
Baseline NIHSS	1.09(1.05-1.13)	<0.001	1.07(1.02-1.11)	0.0018	1.07(1.02-1.11)	0.0017
TOAST classification						
Large artery atherosclerosis	3.71(1.06-13.04)	0.041				
Cardio embolism	4.71(1.37-16.23)	0.014				
Others	3.57(0.97-13.07)	0.055				
Small-artery occlusion	Reference		Reference		Reference	
Occlusion site						
Carotid occlusion	3.61(1.89-6.90)	<0.001	1.88(0.87-4.06)	0.11	1.71(0.78-3.77)	0.18
MCA occlusion (M1-M2)	1.04(0.53-2.03)	0.908	0.62(0.27-1.40)	0.25	0.59(0.26-1.35)	0.21
No record or other	Reference		Reference		Reference	
Endovascular treatment	1.36(0.70-2.63)	0.359	1.16(0.50-2.68)	0.73	1.27(0.55-2.91)	0.57
Intravenous thrombolysis	0.56(0.23-1.36)	0.196	0.53(0.21-1.36)	0.19	0.53(0.20-1.36)	0.18
Antihypertensive therapy	0.82(0.47-1.43)	0.475				
Insulin	1.45(0.70-3.00)	0.321				
Oral hypoglycemic agents	0.87(0.39-1.94)	0.731				
Antiplatelet	0.32(0.16-0.63)	0.001				
Statin	0.48(0.25-0.94)	0.032				
mTICI (2b/3)	0.77(0.14-4.28)	0.761				
RPG	1.04(0.96-1.12)	0.314	1.04(0.93-1.16)	0.45	1.04(0.93-1.16)	0.47
SHR(per 0.1-point increases）	1.21(1.11-1.31)	<0.001	1.13(1.03-1.25)	0.01		

Variables	Univariate analysis OR (95%CI)	*P* value	Multivariate analysis OR (95%CI), model 1	*P* value	Multivariate analysis OR (95%CI), model 2	*P* value
SHR (≥1.25)	4.64(2.59-8.31)	<0.001			2.79(1.42-5.49)	0.003

OR, odds ratio; CI, confidence interval; TIA. transient ischemic attack; NIHSS, the National Institutes of Health Stroke Scale; TOAST, Trial of Org 10172 in Acute Stroke Treatment; MCA, middle cerebral artery; mTICI. modified Thrombolysis in Cerebral Infarction; RPG: random plasma glucose; SHR. stress hyperglycemia ratio

Supplementary Table 4. Predictive performance of dichotomized SHR (⩾1.25 versus < 1.25) on training dataset and validation dataset

	moderate-to-severe CED	90-day poor functional outcome (mRS >2)	90-day death
Training dataset			
Sensitivity (%)	50.59	29.21	42.42
Specificity (%)	89.75	92.97	86.29
PPV (%)	56.58	81.94	38.89
NPV (%)	87.31	54.6	87.94
Accuracy (%)	81.57	59.69	78.81
Validation dataset			
Sensitivity (%)	49.09	41.41	44.44
Specificity (%)	76.70	82.88	73.77
PPV (%)	39.71	68.33	20
NPV (%)	82.82	61.33	90
Accuracy (%)	70.13	63.33	70

CED: Cerebral edema; PPV: Positive Predictive Value; NPV: Negative Predictive Value

1.1 Supplementary Figure

Supplementary Figure 1. Association between the stress hyperglycemia ratio (SHR) and moderate-to-severe cerebral edema (training dataset).

	SHR<1.25 n(%)	SHR≥1.25 n(%)	OR(95%CI)	P for interaction
**Overall**				
**Crude**	**42(12.7)**	**43(56.6)**	**8.97(5.14, 15.65)**	
**Adjusted**				
**Age(≥65)**				**0.64**
No	14(12.1)	10(55.6)	10.03 (2.40, 41.95)	
Yes	28(13)	33(56.9)	6.20 (2.80, 13.74)	
**NIHSS(≥15)**				**0.65**
No	19(8)	16(41)	4.29 (1.57, 11.77)	
Yes	23(24.7)	27(73)	6.60 (2.36, 18.51)	
**Diabetes**				**0.21**
No	40(15)	31(60.8)	5.19 (2.42, 11.10)	
Yes	2(3.1)	12(48)	25.41 (2.25, 287.27)	
**EVT**				**0.53**
No	33(11.6)	30(54.5)	5.36 (2.21, 12.96)	
Yes	9(19.1)	13(61.9)	11.39 (2.64, 49.08)	
**IVT**				**0.27**
No	31(10.9)	39(59.1)	8.47 (3.76, 19.08)	
Yes	11(23.4)	4(40)	2.83 (0.36, 22.47)	

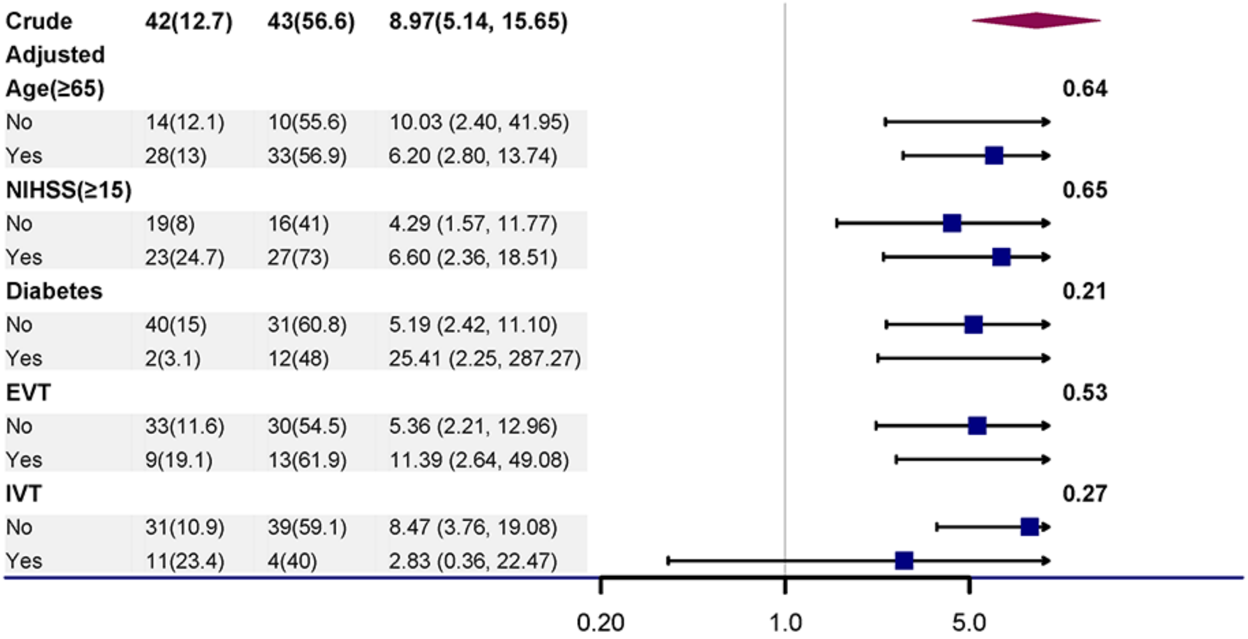

The following covariates expect for the stratified variables were adjusted as in model 2(Supplementary table 2): age, the National Institutes of Health Stroke Scale (NIHSS) score, diabetes, endovascular treatment, intravenous thrombolysis, occlusion site, and admission plasma glucose. SHR: stress hyperglycemia ratio, OR: odds ratio, CI, confidence interval; EVT: endovascular treatment, IVT: intravenous thrombolysis

Supplementary Figure 2. Association between the stress hyperglycemia ratio (SHR) and 90-day poor functional outcome (training dataset)

	SHR<1.25 n(%)	SHR≥1.25 n(%)	OR(95%CI)	P for interaction
**Overall**				
**Crude**	**143(45.4)**	**59(81.9)**	**5.46(2.88-10.35)**	
**Adjusted**				
**Age(≥65)**				**0.33**
No	41(37.6)	10(62.5)	2.70 (0.78, 9.38)	
Yes	102(49.5)	49(87.5)	4.85 (1.79, 13.17)	
**NIHSS(≥15)**				**0.83**
No	82(36.6)	25(71.4)	3.69 (1.53, 8.87)	
Yes	61(67)	34(91.9)	3.88 (0.90, 16.69)	
**Diabetes**				**0.62**
No	121(48)	40(81.6)	3.31 (1.36, 8.08)	
Yes	22(34.9)	19(82.6)	6.20 (1.19, 32.21)	
**EVT**				**0.027**
No	122(45.5)	41(78.8)	2.34 (0.99, 5.53)	
Yes	21(44.7)	18(90)	23.07 (2.88, 184.84)	
**IVT**				**0.75**
No	126(46.7)	52(83.9)	3.78 (1.56, 9.15)	
Yes	17(37.8)	7(70)	4.16 (0.82, 20.97)	

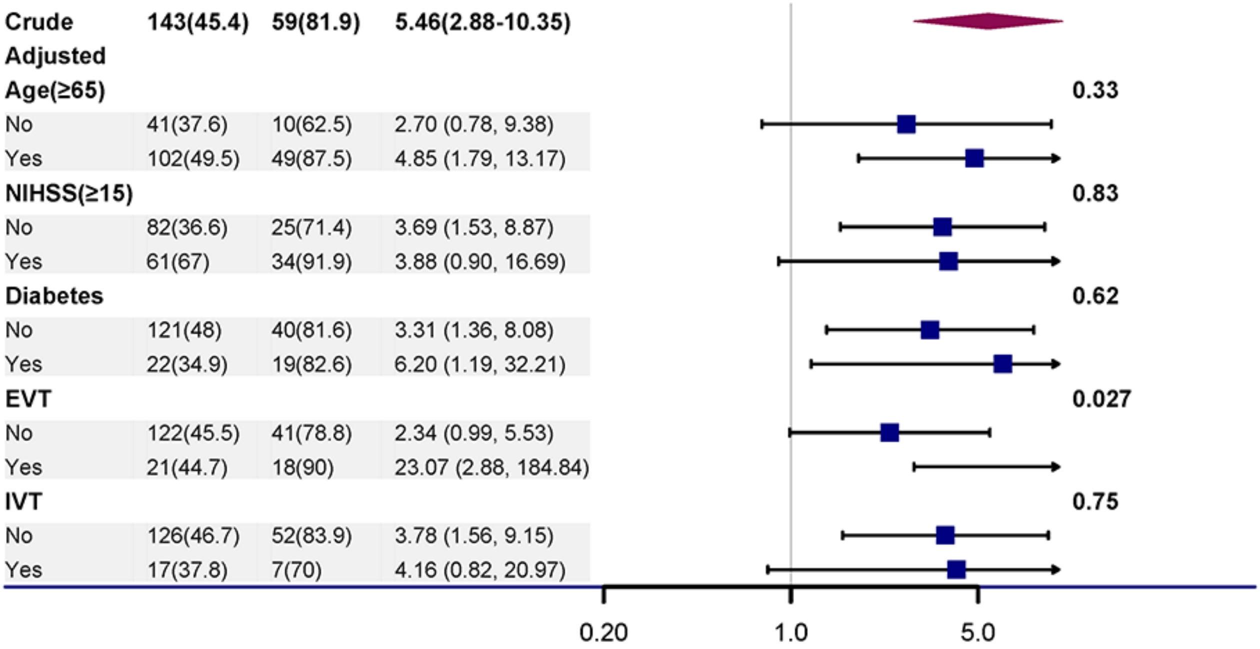

The following covariates expect for the stratified variables were adjusted as in model 2(table 3): age, the National Institutes of Health Stroke Scale (NIHSS) score, diabetes, endovascular treatment, intravenous thrombolysis, occlusion site, previous stroke/transient ischemic attack, and admission plasma glucose. SHR: stress hyperglycemia ratio, OR: odds ratio, CI, confidence interval; EVT: endovascular treatment, IVT: intravenous thrombolysis

Supplementary Figure 3. Association between stress hyperglycemia ratio (SHR) and 90-day death (training dataset)

	SHR<1.25 n(%)	SHR≥1.25 n(%)	OR(95%CI)	P for interaction
**Overall**				
**Crude**	**38(12.1)**	**28(38.9)**	**4.64(2.59,8.31)**	
**Adjusted**				
**Age(≥65)**				**0.067**
No	6(5.5)	6(37.5)	25.78 (3.17, 209.68)	
Yes	32(15.5)	22(39.3)	1.99 (0.92, 4.28)	
**NIHSS(≥15)**				**0.72**
No	21(9.4)	10(28.6)	2.75 (1.03, 7.35)	
Yes	17(18.7)	18(48.6)	2.28 (0.84, 6.17)	
**Diabetes**				**0.68**
No	30(11.9)	19(38.8)	3.36 (1.52, 7.43)	
Yes	8(12.7)	9(39.1)	1.75 (0.36, 8.42)	
**EVT**				**0.67**
No	32(11.9)	20(38.5)	2.38 (1.07, 5.33)	
Yes	6(12.8)	8(40)	4.31 (1.00, 18.67)	

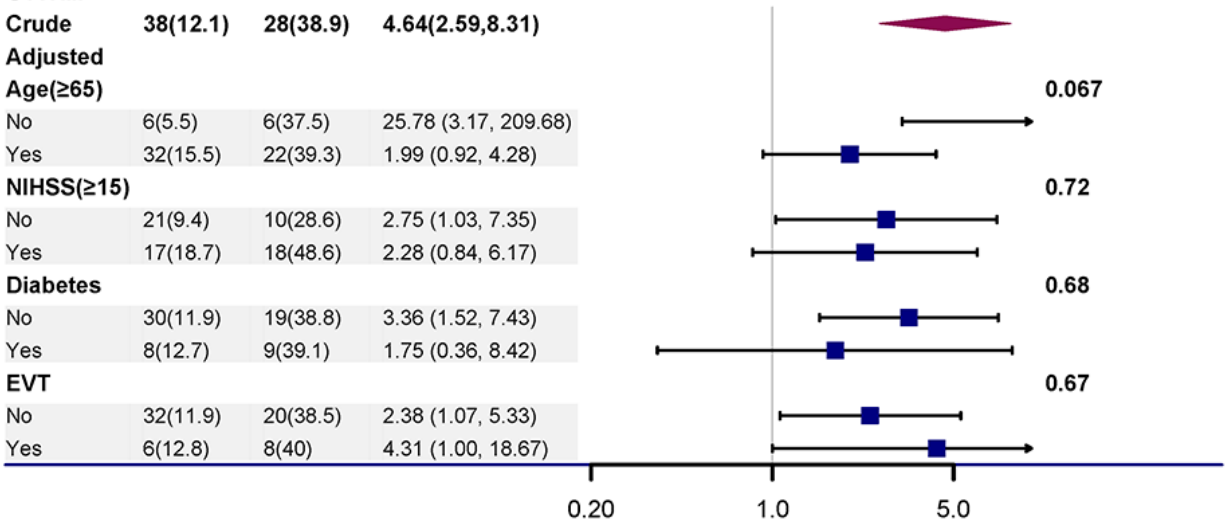

The following covariates expect for the stratified variables were adjusted as in model 2(Supplementary table 3): age, the National Institutes of Health Stroke Scale (NIHSS) score, diabetes, endovascular treatment, intravenous thrombolysis, occlusion site, previous stroke/transient ischemic attack, and admission plasma glucose. SHR: stress hyperglycemia ratio, OR: odds ratio, CI, confidence interval; EVT: endovascular treatment
